# T-cell immune adaptor SKAP1 regulates the induction of collagen-induced arthritis in mice

**DOI:** 10.1016/j.imlet.2016.04.007

**Published:** 2016-08

**Authors:** Xin Smith, Alison Taylor, Christopher E. Rudd

**Affiliations:** Cell Signalling Section, Department of Pathology, Tennis Court Road, University of Cambridge, Cambridge CB2 1Q, UK

**Keywords:** T-cells, SKAP1, SKAP-55, Adaptor, Murine autoimmune arthritis

## Abstract

SKAP1 is an immune cell adaptor that couples the T-cell receptor with the ‘inside-out’ signalling pathway for LFA-1 mediated adhesion in T-cells. A connection of SKAP1 to the regulation of an autoimmune disorder has not previously been reported. In this study, we show that Skap1-deficient (*skap1-/-*) mice are highly resistant to the induction of collagen-induced arthritis (CIA), both in terms of incidence or severity. *Skap1-/-* T-cells were characterised by a selective reduction in the presence IL-17+ (Th17) in response to CII peptide and a marked reduction of joint infiltrating T-cells in *Skap1-/-* mice. SKAP1 therefore represents a novel connection to Th17 producing T-cells and is new potential target in the therapeutic intervention in autoimmune and inflammatory diseases.

## Highlights

• Skap1-deficient (*skap1-/-*) mice are resistant to the induction of collagen induced arthritis (CIA).
• *Skap1-/-* mice show a reduction in presence of IL-17+ (Th17) T-cells in response to CII peptide.
• No effect was seen on the production of other cytokines such as IL-10.
• Our findings implicate SKAP1 as a novel upstream regulator murine autoimmune arthritis.

## 1 Introduction

Rheumatoid arthritis (RA) is a chronic inflammatory disease that affects joints, causing cartilage and bone destruction. The disease can be induced by the immunization of susceptible mice with type II collagen (CII) leading to humoral and cell-mediated responses [1], [2]. Arthritic severity in murine arthritis is correlated with autoantibody responses to CII [3]. Further, CD4+ T cell involvement in Rheumatoid arthritis (RA) has been inferred from linkage to HLA-DR alleles and the presence of activated CD4+ T cells in inflamed joints [4]. Despite the success of Tumour necrosis factor-alpha (TNF-α) blocking biologics in treating RA [2], [5], novel therapies are needed to modulate the underlying immune response [2], [6]. Murine models have been used in the pre-clinical testing of drugs such as TNF-α blockade [5], [7].

LFA-1 (lymphocyte function-associated antigen; αLβ2) antagonists also reduce inflammation in murine arthritis [8]. LFA-1 on T cells binds ICAM-1/3 on antigen presenting cells [9], [10], and its activation involves GTP-binding protein Rac-1 [11], [12], [13], [14], [15] and immune cell adaptors in T-cells termed ADAP (adhesion and degranulation-promoting adaptor protein; also FYB: Fyn binding protein) and SKAP1 (Src-kinase-associated phosphoprotein1 or SKAP-55) [16]. SKAP1 has a pleckstrin-homology domain, a C-terminal SH3 domain and tyrosine residues [17], [18] and regulates integrin clustering and adhesion [12], [13], [16], [19], [20] via the Rap1 binding to its partner, RAPL [16], [21], [22].

Despite its importance in T-cell function, a connection of SKAP1 to an autoimmune disorder has not been reported. Here, we show that Skap1-deficient mice are resistant to the induction of CIA, as characterised by reduced pro-inflammatory cytokine IL-17. Our finding indicates for the first time a role for SKAP1 in regulating inflammatory arthritis and suggests that the adaptor might serve as a novel therapeutic target.

## 2 Results and discussion

### 2.1 *Skap1-/-* mice are less susceptible to collagen-induced arthritis (CIA)

**Fig. 1:**
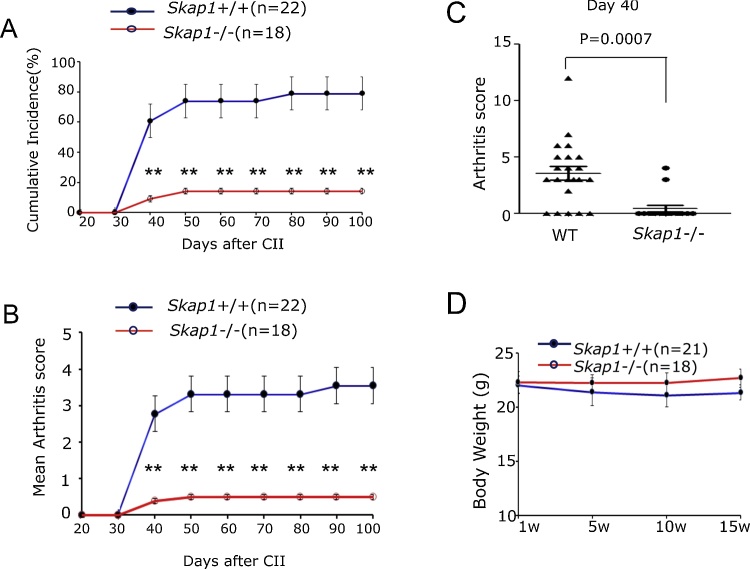
*Skap1*−/− mice are resistant to collagen induced arthritis. Collagen induced arthritis (CIA) was induced in wild type mice and *Skap1*−/− mice as described in Section 3. Panel A: *Skap1*−/− mice show a reduced incidence of inflammation. Panel B: *Skap1*-/- mice show a reduced mean arthritis score. Reduced clinical scores in *Skap1*−/− mice from day 20–90. The Data shown in Panel A and B are from four pooled experiments. The presented results are the mean ± SEM.* p < 0.05, **p < 0.01. Panel C: Dot plot of individual arthritis scores of mice at day 40. *Panel D*: The body weight of *Skap1−/−* and WT mice from week 1–15. No difference in body weights was observed.

To assess the role of SKAP1 in the induction of CIA, *Skap1*+/+ and *Skap1*-/- mice were injected intradermally (i.d.) at two sites into the base of the tail with a chicken type II (CII) in an emulsion of complete Freund adjuvant (CFA) and monitored for signs of disease over 100 days (see Section 3). CII collagen can induce arthritis in C57BL/6 mice with incidences of 50–70% [23], [24], [25], [26]. *Skap1*+/+ and *Skap1*-/- mice have the same genetic background, and in many cases, were littermates. We found that CII collagen induced arthritis in B6 mice with a maximum cumulative incidence of 70% and mean day of onset between days 30 and 40 after primary immunization, as reported [26] (Fig. 1A). By contrast, *Skap1-/-* mice showed a much lower cumulative incidence of 10% (n = 18–22). The mean arthritic score was also lower in *Skap1-/-* mice from day 50–80 with a value of 3.7 for *Skap1*+/+ mice and 0.4 for *Skap1-/-* mice (Fig. 1B). A dot plot of different mice at day 40 showed the range of responses for *Skap1*+/+ vs. −/- mice with a mean that was consistently lower for *Skap1-/-* mice (p value = 0.0007) (Fig. 1C). No difference was noted in the body weight of *Skap1*+/+ and *Skap1*-/- mice (Fig. 1D). These data showed that the loss of SKAP1 conferred relative resistance of mice to the induction of arthritis.

**Fig. 2:**
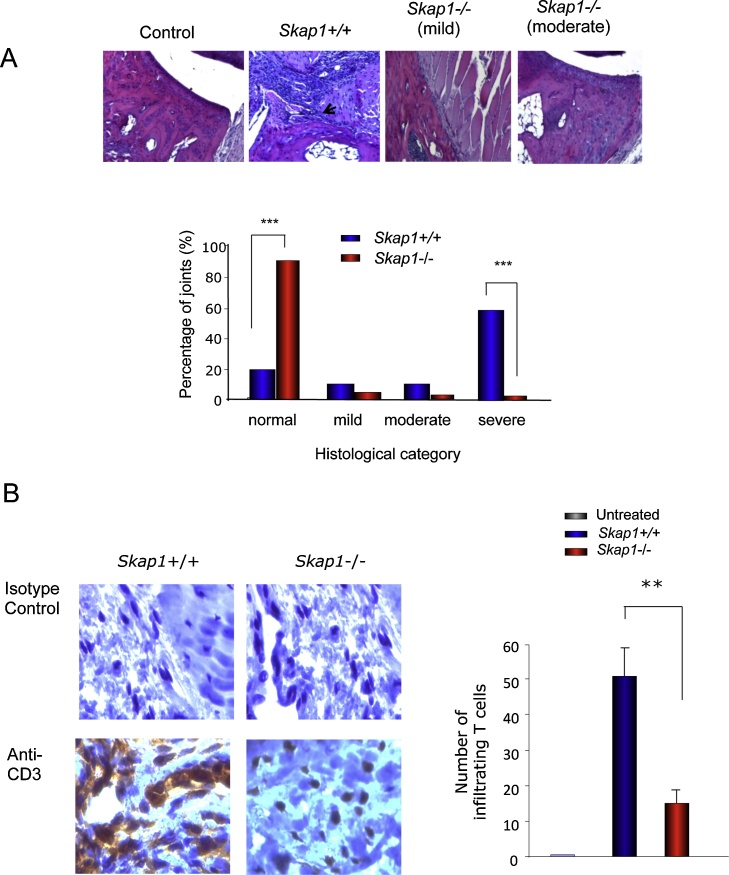
Joint histology of *Skap1−/*− vs *Skap1*+/+ (WT) mice. Panel A: Joint histology. *Upper panels:* Histological features of CIA. Sections of an untreated control (left panel), severely arthritic Skap1+/+ (middle left), mild arthritic CIA-immunized *Skap1−/−* mouse (middle right) and moderately diseased *Skap1−/−* mouse (right panel) (×200 magnification). *Lower panel*: Histogram of percentage of joints. SKAP1-/- (n = 504 joints) and WT (n = 556 joints) mice in each group. Data are from four pooled experiments. ***P < 0.001 compared with the same histological grade in WT mice. (n = 18–22). Panel B: *Skap1−/− mice show a reduced numbers of infiltrating T-cells in joints.* Immunoperoxidase staining of the tissue sessions with an anti-CD3 Ab. Dark brown cells indicate CD3-positive T cells (×40). Upper panel: images of stained sections; lower panel: histogram showing the number of T-cells in joints on 0–50 scale. At least five areas from each specimen were chosen to determine the numbers of T cell infitrating in each specimen. T cells infiltration of the synovial area of *Skap-1-/-*mice was significantly reduced, compared with WT mice. P = 0.0005.

A histological assessment of joints showed that 60% of *Skap1+/+* mice showed severe pathology, 10% moderate, 10% mild and 20% normal histology (Fig. 2A, upper and lower panels). Severely affected joints showed inflammatory cell infiltration and invasive pannus tissue formation associated with bone and cartilage degradation. By contrast, *Skap*–/– mice showed 85% in the normal category when compared to the WT mice (*P* < 0.001). The remaining 5% showed either a mild and moderate pathology pattern. Overall, these findings showed that SKAP1 deficiency provides protection against the induction of CIA. We also examined the presence of T-cells in the joints *Skap1*-/- and +/+ mice in response to CII. CD3+ T cells were subjected to in situ staining of the joint area and revealed a major reduction in the infiltration of T-cells in Skap1−/− mice (Fig. 2B; T-cells seen in brown color). Mice were immunized with CII and boosted at day 21, the mice were sacrified at day 40, and the joints were sectioned and stained with antibody. In a given area, 52 CD3+ T-cells were detected in skap1+/+ mice, and only 16 in skap1-/- mice (right histogram). There were no detectable T-cells in non-diseased mouse joints.

### 2.2 Reduced expression of IL-17 in T-cells of *Skap1*-/- mice

**Fig. 3:**
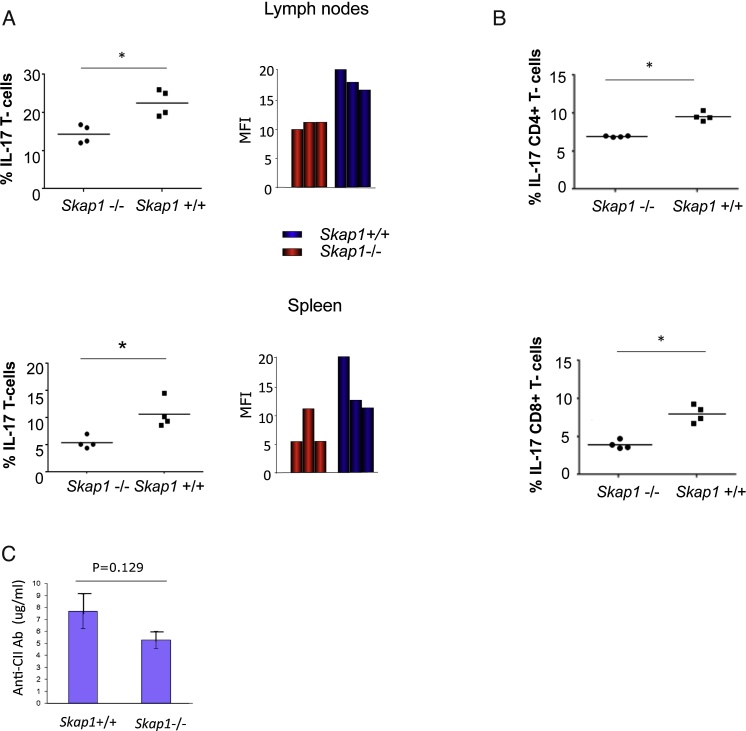
Cellular and humoral responses to CII in mice. T-cells were removed from mesenteric lymph nodes at day 14 following CII injection and assessed for the various cytokines by intracellular staining and flow cytometry. Panel A: The number of CD3+ IL-17 expressing T-cells is reduced in *Skap1-/-* mice in response to CII. Upper panels: lymph nodes; lower panels: spleen. Left panels: Percentage of cells; right panels: mean fluorescent intensity (MFI). Panel B: IL-17 expressing CD4 and CD8 cells reduced in *Skap1-/-* mice in response to CII. Upper panels: IL-17 expression in CD4+ T-cells; lower panels: IL-17 expression in CD8+ T-cells. Panel C*:* Serum anti-CII IgG levels. Circulating levels of CII-specific IgG were determined in individual sera from *SKAP1*-/- (n = 18) and WT (n = 22) mice at 100 days after primary immunization with CII. Results show the mean± SEM (n = 3).) *p < 0.05, **p < 0.01.

To investigate cytokine responses, inguinal LNs and spleen were harvested on day 14 (early arthritis) post primary CII immunization followed by intracellular staining for various cytokines (Fig. 3). When gated on CD3, *Skap1-/-* LNs showed a significantly lower percent of IL-17 expressing cells with 15.5% relative to 22.6% for *Skap1+/+* cells (Fig. 3A, upper left panel). The MFI for *Skap1-/-* T-cells was also lower (upper right panel). Similarly, only 5.6% of CD3 positive splenic *Skap1−/−* T-cells were positive relative to 10.7 for *Skap1+/+* T-cells (lower left panel). The MFI was 6.2 for *Skap1-/-* spleen T-cells relative to 11.7 for wild-type cells (lower right panel) (n = 5). Likewise, when gated for CD4, *Skap1-/-* CD3+ spleen T-cells showed a lower percentage of IL-17 positive cells with 7.2% versus 10.2% for *Skap1+/+* T-cells (Fig. 3B, upper panel; Fig. S1). When gated for CD8, 4.3% of *Skap1-/-* CD3+ T-cells expressed the cytokine relative to 9.0% of CD3+ *Skap1+/+* T-cells. These data showed that *Skap1-/-* T-cells have a lower prevalence of IL-17 expression in response to CII immunization. Surprisingly, the expression of other inflammatory or inhibitory cytokines such as IL-10, TNF-α, IFN-β, and IL-2 was unaffected by the loss of SKAP1 (Fig. S2A). No difference was noted in the expression of surface receptors such as the differentiation marker CD62L or inhibitory co-receptors such as PD-1 and LAG-3 (Fig. S2B). A difference was noted for CTLA-4 expression in T-cells from spleen but not lymph nodes.

As an additional control, anti-serum anti-C11 antibody levels were assessed by ELISA (Fig. 3C). Blood was collected from the retro-orbital sinus at day 40 post-primary immunization, while bound anti-collagen IgG was detected with HRP-labelled goat anti-mouse IgG. *Skap1+/+* mice (WT) showed an average of 7.5 μg/ml of anti-CII antibodies, while a mean was reduced to 5.4 μg/ml in *Skap1-/-* mice as averaged over 3 experiments. No significant difference in antibody production was seen between Skap1−/− and WT mice, consistent with the report that Th17 dervived IL-17 is dispensible for antibody production [27].

Overall, our findings identify SKAP1 for the first time as a key regulator of CII induced RA in mice. *Skap1*-deficient mice were markedly resistant to the induction of CIA as characterised by a reduction in the key pro-inflammatory cytokine IL-17. Both the number of Th17 cells and the production of IL-17 were significantly reduced, an effect that would be expected to reduce the inflammatory response. This is also the first reported connection between SKAP1 and the generation of Th17 cells. The ability of SKAP1 to mediate inflammatory responses is consistent with its role in mediating T-cell binding to antigen presenting cells and ensuring oprtimal responses to antigen [12]. The exact integrin targeted by Skap1 in resistence is not clear since the activation of both β1 and β2 integrins is impaired in *skap1−/*− T-cells [19], [20]. Leukocyte recruitment to sites of inflammation is a multistep process that involves the multiple adhesion molecules and their ligands on leukocytes and the inflamed tissue. Antibody blocking studies suggest that the LFA-1 I-domain mediates a critical interaction in synovial inflammation, either to counterreceptors ICAM-1 (CD54) or JAM-A in the synovial [28]. Disease onset and initial severity was affected in ICAM-1 null mice, but not ICAM-2 null mice. Another report has shown a marked reduction in acute leukocyte recruitment in CD18- and CD11a-deficient mice in peritoneal inflammation, but not in mice lacking CD18 [29]. Our findings are therefore consistent with a role for SKAP1 in facilitating the activation of LFA-1 adhesion in inflammation. It provides an alternative target to anti-LFA-1 in therapeutic intervention in inflammatory arthritis.

Our findings are also compatible with the finding that SKAP1 is needed primarily in responses to lower affinity or dose antigen [30], [31]. We previously showed that high TCR occupancy with anti-CD3 can over-ride the dependency of LFA-1 activation on the SKAP1 adaptor [19]. At the same time, it was surprising that other cytokines such as IL-10 were not affected. The rationale for this selectivity is unclear, but may related to additional roles of SKAP1 in the inhibition of the p21-ERK pathway [32], [33] and in ‘outside-in’ signalling via integrins [13], [34]. While the full range of mediators affected by these processes are unknown, the balance of positive and negative signals could alter the threshold of signalling favouring the induction of one cytokine vs. another in different conditions. The potency of the effect of the loss of SKAP1 also suggests that its function cannot be substituted by related Skap2 (Hom or related) [35]. Overall, our findings suggest that SKAP1 may be an attractive target for therapeutic intervention in autoimmune and inflammatory diseases.

## 3 Materials and methods

### 3.1 Collagen-induced arthritis (CIA)

C57BL/6J mice from Jackson laboratories (Bar Harbor, ME) while *skap1-/-* mice were derived as decribed in Refs. [19], [36]. Chick type II collagen, CII (Sigma) in Complete Freund adjuvant (CFA) was injected intradermally (i.d.) at two sites into tail base (100 μl emulsion with 100 μg CII and 250 μg *Mycobacterium tuberculosis*) [7]. Injections were repeated on day 21 and joints were assessed for swelling and restriction. Each paw was scored (0 indicates normal; 1, mild swelling; 2 extensive swelling; and 3, joint distortion and/or rigidity; maximum score per mouse, 12). Joint histology was examined by joints (knee, elbow, ankle and wrist) being harvested, fixed in 10% formaldehyde/PBS forand decalcified using EDTA for 4 weeks. Specimens were cut longitudinally to the midline, and 5 μm sections mounted for staining with haematoxylin and eosin. These sections were graded in severity from 0 (normal) to 5 (severe) for five components, including joint exudate, synovitis, panus formation, cartilage degradation and bone degradation. Based on the histological scores, joints were classified as demonstration inflammatory arthritis if there was an exudate score of 1 or more and a synovitis score of 2 or more. Destructive arthritis was defined for joints that scored 2 or more for pannus or 1 or more for either cartilage degradation or bone degradation.

T cell infiltration in the joint was determined by immunoperoxidase staining of tissue sections with anti-CD3 (DAKO) for 1 h. DAB staining kit (SK4100, Vector Laboratories) was used for color visualization and counterstaining with hematoxylin and eosin. Five fields were randomly selected for each joint, and the average number of infiltrating T cells was determined by adding the total number of T cells, then dividing by five to obtain the number of infiltrating T cells per filed of each joint.

### 3.2 CII-specific and cytokine responses

Isotype-specific anti-collagen Abs from retro-orbital sinus blood was determined by ELISA on Immulon II plates (Dynex Technologies, Chantilly, VA). Bound anti-collagen IgG was detected with HRP-labeled goat anti-mouse IgG (Southern Biotechnology Associates, Birmingham, AL). Absorbance at 450 nm.

For cytokine production, T-cells from inguinal LNs or spleen were harvested at 14 days post immunization followed by intracellular staining for TNFα, IL-2 and IFN-γ and other cytolines (BD). Differences between mice was analyzed by a Mann-Whitney *U* test. All data are represented as mean ± SEM or SD and values of p < 0.05 were considered statistically significant. Flow cytometry was also conducted using antibodies to CD3, CD4, CD44, CD62L (BD, Cell Signal) with Alexa conjugated secondary antibodies (Invitrogen).

## Conflict of interest

The authors have no conflict of interest or competing interests.
