# Supplementary figures and images for: T-cell immune adaptor SKAP1 regulates the induction of collagen-induced arthritis in mice
Source: Immunol Lett. 2016 Aug;176:122–7. doi: 10.1016/j.imlet.2016.04.007 (PMC4965781; doi:10.1016/j.imlet.2016.04.007)

Supplemental Fig. 1

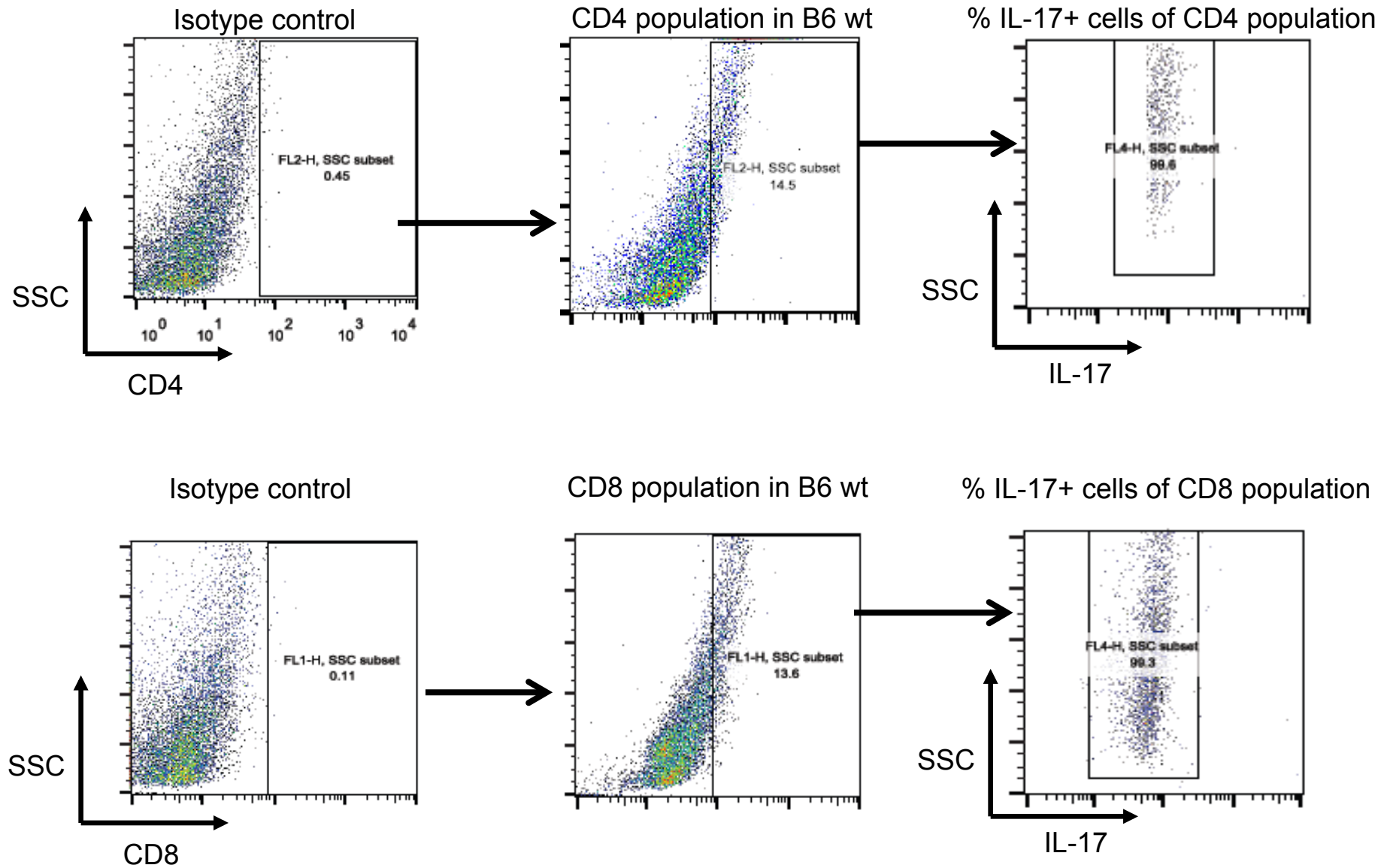

Supplement: Supplementary file 1 [file mmc1.pdf]

Supplemental Figure 2

A

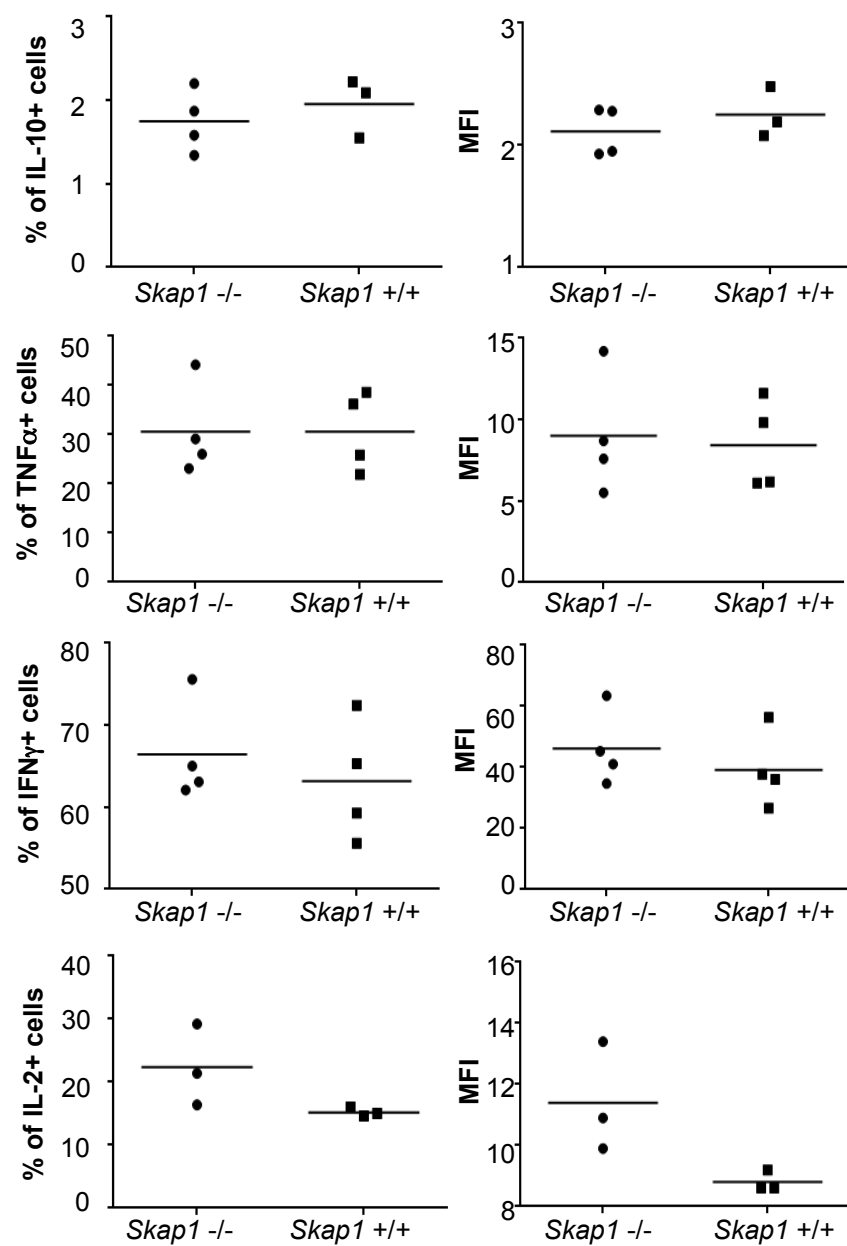

B

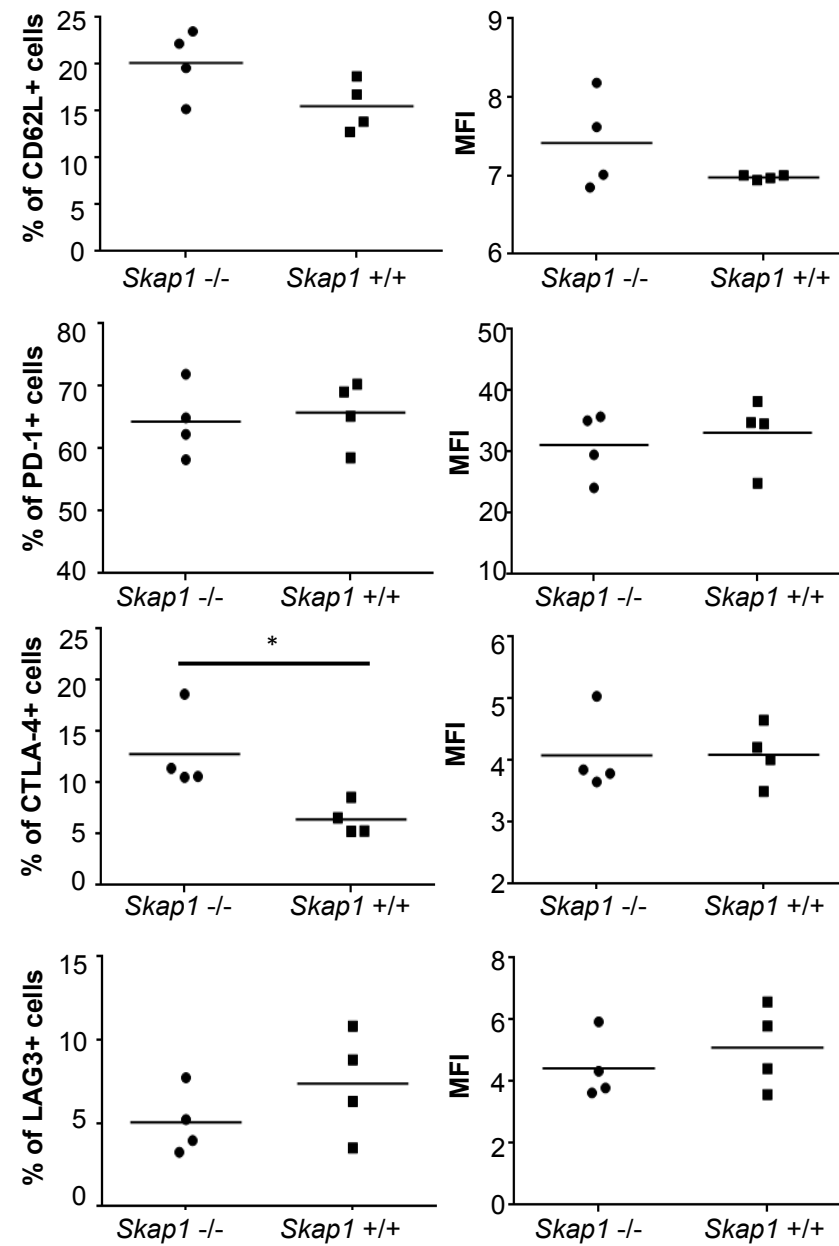

Supplement: Supplementary file 2 [file mmc2.pdf]
