# Supplementary material for: T-cell immune adaptor SKAP1 regulates the induction of collagen-induced arthritis in mice
Source: Immunol Lett. 2016 Aug;176:122–7. doi: 10.1016/j.imlet.2016.04.007 (PMC4965781; doi:10.1016/j.imlet.2016.04.007)
Supplement: Supplementary file 3 [file mmc3.pdf]

Supplementary Figure Legends

SFigure 1: ***Gating of CD4 and CD8 positive T-cells for IL-17 expression by FACs.*** Cells were removed from mesenteric lymph nodes and labelled with anti-CD4 (upper middle panel) or anti-CD8 (lower middle panel) followed by staining with Alexa APC-Fluor 405 conjugated secondary antibodies. Isotype antibody served as a negative control for gating against CD4 and CD8 positive subsets. Directly conjugate anti-IL-17 stained for expression of the cytokine in permeabilised and fixed cells.

SFigure 2: ***Cellular response to CII in mice.*** T-cells from spleen or lymph nodes were removed at day 14 following CII injection and assessed intracellularly for the various cytokines by intracellular staining and flow cytometry. **Panel A:** Comparison of IL-10, TNF-α, IFN-γ and IL-2 expression. T-cells from spleen or lymph nodes (LNs) were removed at day 14 and assessed for expression by flow cytometry. **Panel B:** Comparison of CD62L, PD-1, CTLA-4 and LAG-3 expression. Spleen (left panel) or lymph nodes (right panel). A significant difference was seen CTLA-4 in spleen but not LNs ($p>0.05$).
